# Broadband terahertz time-domain polarimetry based on air plasma filament emissions and spinning electro-optic sampling in GaP

**DOI:** 10.1063/5.0087127

**Published:** 2022-05-03

**Authors:** Kuangyi Xu, Mengkun Liu, M. Hassan Arbab

**Affiliations:** 1 Department of Biomedical Engineering, State University of New York at Stony Brook, Stony Brook, New York 11794, USA; 2 Department of Physics and Astronomy, State University of New York at Stony Brook, Stony Brook, New York 11794, USA

## Abstract

We report on a time-domain polarimetry (TDP) system for generating and detecting broadband terahertz (THz) waves of different polarization angles. We generate THz waves from two-color laser filaments and determine their polarization states with a detection bandwidth of up to 8 THz using a spinning gallium phosphide crystal. The polarization of THz emission can be controlled by adjusting the position and tilt angle of the β-barium borate crystal. We characterize the precision of this system for polarimetric measurements at fixed time delay to be 1.6° and 1.9° for complete time-domain waveforms. We also demonstrate the feasibility of our TDP system by measuring broadband optical properties of anisotropic samples in both transmission and reflection geometries. The THz-TDP technique can be easily integrated in conventional THz time-domain spectroscopy setups using nonlinear crystal detectors.

Polarimetry refers to the measurement of the polarization state of electromagnetic waves transmitted through or reflected by samples. The polarization information of THz waves can be used in different aspects of material characterization such as the ellipsometric measurement of thin-films,^1^ the detection of the birefringence and optical axis of samples,^2^ and the investigation of THz magneto-optic properties of materials.^3^ Compared to optical polarimetry, limited availability of achromatic THz polarization elements has made full polarimetric measurement of broadband THz beams challenging. Polarization-sensitive detection in the THz regime is usually obtained using wire-grid polarizers. Current wire grid polarizers can reach 30–40 dB extinction ratio (ER) at lower THz frequencies (<1 THz).^4,5^ However, the extinction ratio is not constant and is significantly reduced at higher frequencies.^6^ The leakage of high frequency components, hence, becomes an intrinsic error when wire-grid polarizers are used in ultra-broadband THz measurements such as those made using gas plasma filaments.

There are several THz polarimetry techniques that do not rely on the use of additional polarization elements. For example, photoconductive antenna detectors are inherently polarization-dependent, since the antenna gap has a certain orientation. Multi-contact antennas with more than one gap are, thus, fabricated to measure the orthogonal components simultaneously.^7–9^ Another strategy is to use the orientation dependence of the electro-optic (EO) sampling technique to resolve a polarization state of the THz beams. The EO responses in zinc blende crystals have a robust relation with the THz polarization, crystal axis, and polarization direction of the probe beam.^10^ Polarization measurements are, thus, achieved by manually rotating the direction of the probe beam^11^ or the detector crystal.^12^ In order to improve the precision and the speed of this method, automatic control and data acquisition have been introduced to replace the steps of manual rotation. Electronic control has been made by polarization or amplitude modulation of the probe beam.^13,14^ Alternatively, the EO crystal can be oriented to specific angles using a motorized rotary stage.^15^ In addition, we have used a continuously spinning EO crystal at a fixed rotational frequency to extract the polarization angle of the THz waves through the Fourier transform in a lock-in type detection technique.^16,17^ However, the bandwidth of our previous TDP system, which utilized 1-mm-thick ZnTe crystals as the emitter and detector, was limited to approximately 2 THz.

Air plasma driven by two-color laser pulses are able to emit broadband THz waves.^18,19^ In this Letter, we propose a THz-TDP system, which uses a two-color laser filament as the source and a spinning gallium phosphide (GaP) crystal as the detector. Compared to ZnTe, GaP has the advantage of a larger THz detection bandwidth due to the higher phonon mode at 11 THz, yet it also has a weaker EO response due to its smaller nonlinear coefficient.^20^ We show that the system described here can measure the frequency spectrum of polarization angles up to 7 THz with the overall precision of 1.6°. Furthermore, we have demonstrated the feasibility of our system by characterizing two anisotropic samples in the transmission and reflection geometries. Our THz-TDP technique can be implemented readily in standard THz-TDS systems using different combinations of nonlinear crystals.

In our previous report,^17^ we demonstrated the precision of the THz-TDP system in the frequency range of 0.1–1.4 THz. We found that if we rotate the (110)-cut EO crystal with an angular frequency of *ω*, the time-dependent balanced signal ΔI(t) is described by

$$\Delta I(t)\propto E_{\text{THz}}[\frac{1}{2}\text{cos}(\omega t+\beta_{0}+\gamma )+\frac{3}{2}\text{cos}(3\omega t+3\beta_{0}-\gamma )],$$ (1)

where the *x*-direction is defined as the polarization direction of the probe beam, *γ* is the polarization angle of THz waves, and *β*_0_ is the initial angle (at *t *=* *0) of the crystal axis [−110], both with respect to the *x*-axis. The amplitude and phase of the *ω* and 3ω terms in Eq. (1) can be used to extract the THz *E*-field strength *E_THz_* and its polarization angle *γ* simultaneously. A lock-in type instrument was designed to obtain more stable measurements.^17^ In this Letter, we focus on extending the reliable bandwidth of our system to 7 THz without changing the measurement technique.

Figure 1 shows the experimental setup. A two-color laser plasma was created by focusing a 35 fs, 800 nm laser through a 100 μm type-I β-barium borate (BBO) crystal. The amplified laser at the repetition rate of *f *=* *1 kHz is modulated by an optical chopper with the rotation frequency of f/2. The polarization-sensitive detection was based on the electro-optic response of a 300 μm thick (110)-cut GaP crystal. This crystal was mounted on a hollow shaft motor rotating at an angular frequency of ω=2π f/31. The Fourier transform of the balanced detector signal ΔI(t) collected over every two revolutions (i.e., 62 ms) was calculated, and the frequency components at *ω* and 3ω were used to extract the amplitude *E*_THz_ and polarization angle *γ*, following the same procedure described previously.^17^ Figure 2 presents the distribution of the polarization angle *γ* with a fixed polarization state of the THz source over 1000 repeats of extraction. The standard deviation at a constant time delay is calculated to be about 1.6°.

**FIG. 1.**
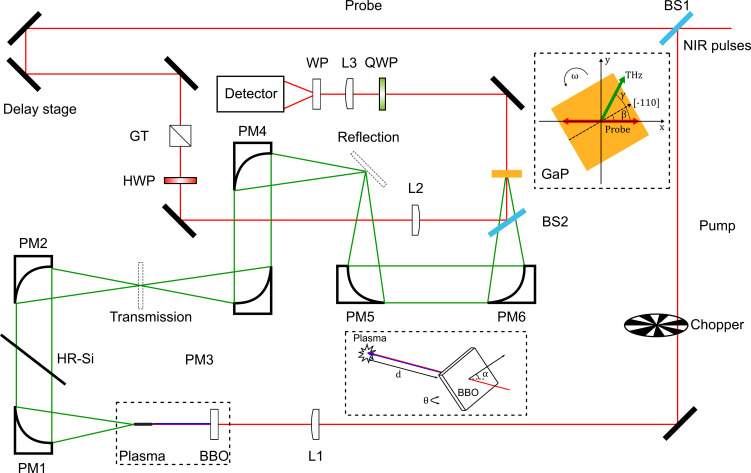
Schematic illustration of the experimental setup. An 800 nm pump beam is focused through a BBO crystal to create a plasma that radiates THz fields. The GaP detector is rotating at a constant frequency *ω* and produces time-dependent balanced signals ΔI(t). Subsequently, the ampitude and angle *γ* of the THz electric field vector are extracted from the Fourier transform of ΔI(t). Inset: manipulation of polarization for the THz source by adjusting the BBO crystal.

**FIG. 2.**
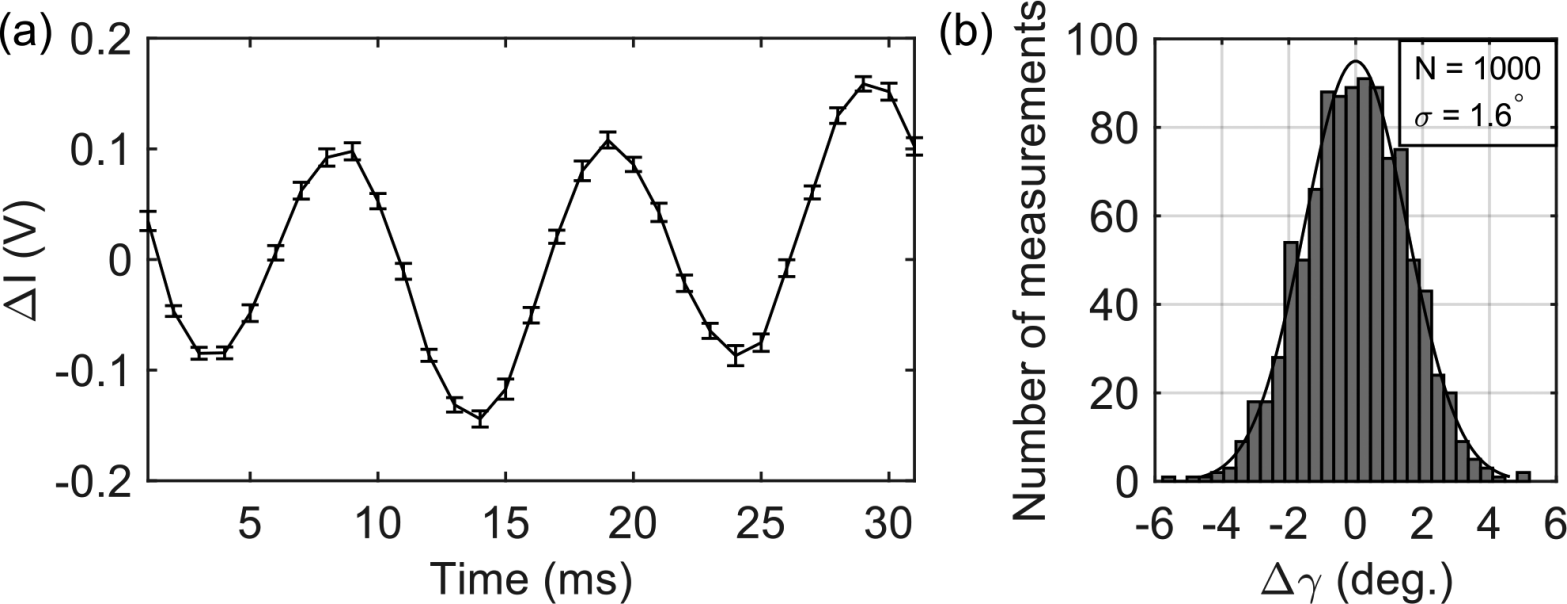
(a) The balanced signal ΔI induced by the THz E-field as a function of time when the GaP detector spins with the constant period of 31 ms. We used *N *=* *1000 measurements to calculate the mean value and the standard deviation of ΔI. (b) The distribution of the E-field polarization angle *γ*. Δγ denotes the deviation from the mean value $\bar{\gamma }$.

Subsequently, we obtained the entire THz waveform as a function of the time delay. At each time-delay *t*_0_, the amplitude *E*_THz_ and argument angle *γ* of $\vec{E}(t_{0})$ were determined using the procedure above. Dried nitrogen gas was purged into the setup box to reduce the absorption of water vapor and enhance the signal bandwidth in the frequency domain. A typical time-domain signal is shown in Fig. 3(a) by averaging five repeated reference measurements. The corresponding frequency spectra in Fig. 3(b) show a maximum electric-field signal-to-noise ratio of 35 dB around 2 THz with a noise-equivalent bandwidth of 8 THz in both the *x*- and *y*-components. We can describe the THz waveform $\vec{E}(t)$ by the azimuthal angle Ψ and phase difference Δ of the polarization ellipse

$$\frac{{\tilde{E}}_{y}(\omega )}{{\tilde{E}}_{x}(\omega )}=\text{tan}\;\Psi e^{i\Delta },$$ (2)

where ${\tilde{E}}_{x}(\omega )$ and ${\tilde{E}}_{y}(\omega )$ are the complex frequency spectrum of the *x*- and *y*-components of $\vec{E}(t)$, respectively. The standard deviation of Ψ using the measurements in Fig. 3(b) was less than 1.9° between 0.7 and 7 THz (not shown), which shows the reliability of high-frequency polarimetric measurements using this technique.

**FIG. 3.**
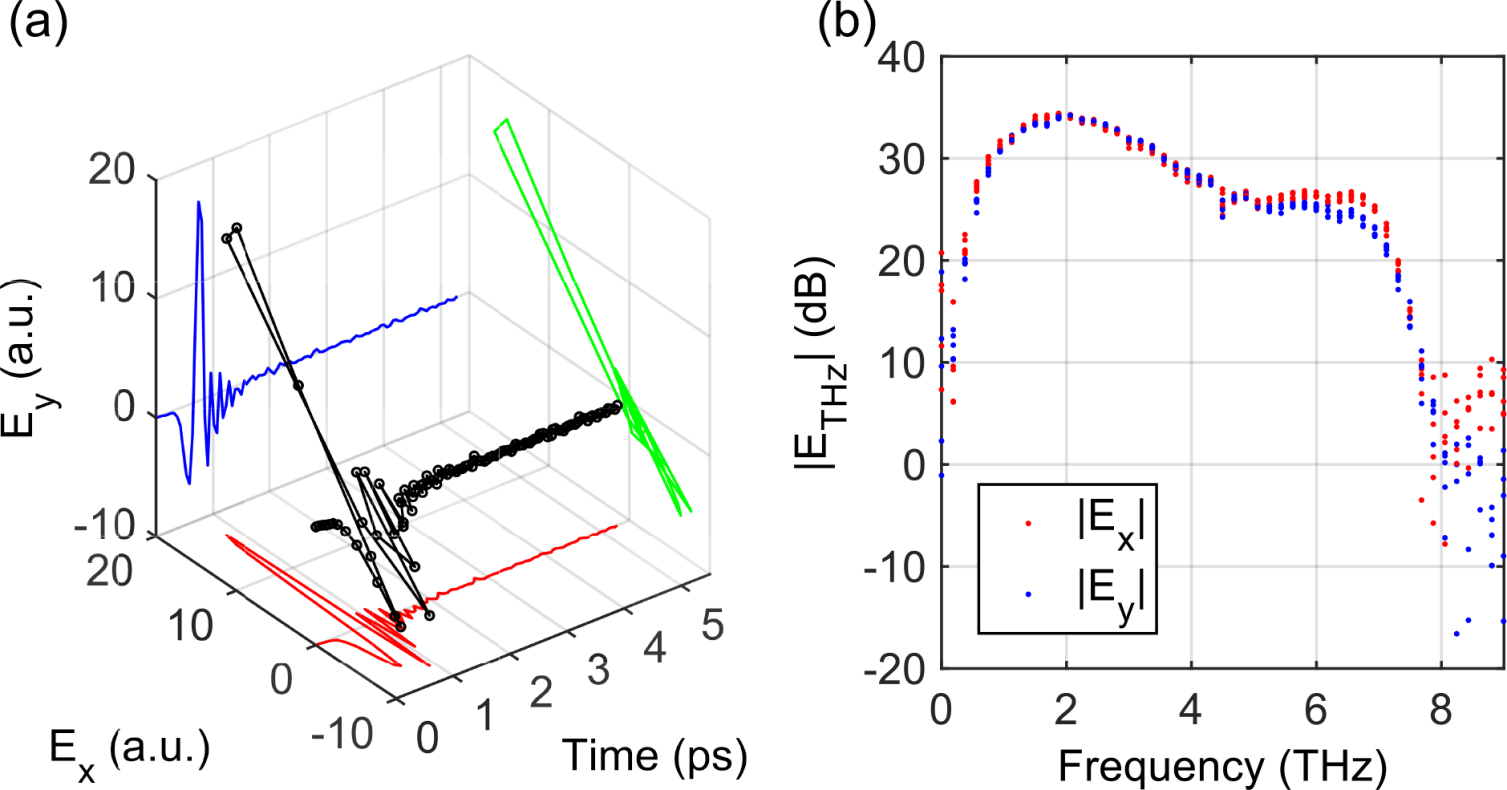
(a) The 3D temporal waveform of a typical THz pulse generated from a plasma filament. (b) The corresponding frequency spectrum of the *x*- and *y*-components.

Controlling the polarization states of THz emission from air plasma is necessary for the complete characterization of the Jones matrices. Here, the polarization control was achieved by adjustments to the BBO crystal. Previously, it is shown that the polarization of THz emission is sensitive to the phase difference between the fundamental and second harmonic fields.^18,19,21,22^ We varied this phase difference by translating the position of the BBO crystal with respect to the plasma. The THz polarization continuously rotates as the distance between BBO and the plasma is changed in the range of 4.9–7.5 cm by a 0.2 cm interval. However, the change in the BBO position results in an oscillation in the maximum THz amplitude $\left|E_{\text{THz}}\right|$. This oscillation can be reduced by adjusting the BBO tilt angle *θ*. Figure 4(a) presents the $\left|E_{\text{THz}}\right|$ as a function of the BBO position at four representative tilt angles *θ*. It can be noted that for θ=−0.15°,0.45°,0.75°, and 1.20°, the ratio between the maximum and minimum $\left|E_{\text{THz}}\right|$ in the sinusoidal curves was 4.60, 2.52, 1.98, and 1.74, respectively. Figure 4(b) shows the resultant polarization states for the two cases where θ=1.2° and −0.15°. In our setup, for conventional TDS measurement that only requires one polarization component, we can use θ=−0.15° to achieve a higher dynamic range. For THz-TDP measurements, however, we can use θ=1.20° to work with different THz polarization angles without dramatic reduction in the precision of polarization detection.

**FIG. 4.**
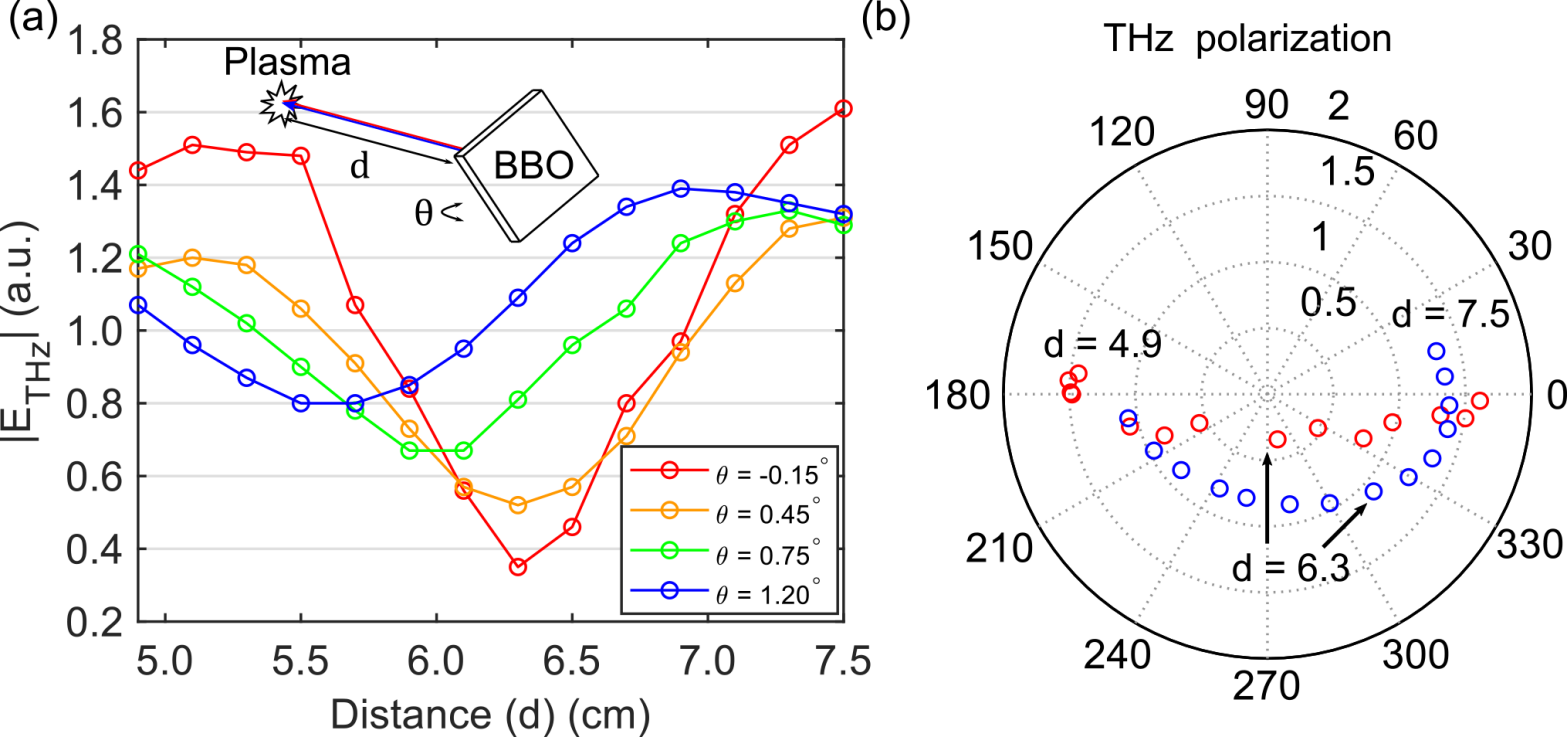
(a) The relation between the THz amplitude and BBO-to-plasma distance *d* under different BBO tilt angles *θ*. (b) The relation between the THz electric vectors and *d* obtained when θ=1.2° (blue) and θ=−0.15° (red).

The amplitude modulation through *θ* is explained by the two-dimensional transverse electron current in the plasma.^21,22^ The laser field *E_L_* in the plasma at time *t* is given by

$$\begin{array}{cc} E_{L}(t)=E_{\omega }[\text{cos}(\omega t)\;\text{cos}\;\alpha \hat{e}+\text{cos}(\omega t+\Gamma )\;\text{sin}\;\alpha \hat{o}]\\ & +E_{2\omega }\;\text{cos}(2\omega t+\varphi )\hat{e}, \end{array}$$ (3)

where $E_{\omega }$ and $E_{2\omega }$ are the amplitudes of the fundamental and the second harmonic beam, respectively, $\hat{e}$ and $\hat{o}$ are the unit vectors of the extraordinary and ordinary crystalline axes of the BBO crystal, respectively, Γ is the phase retardation between $\hat{e}$ and $\hat{o}$ directions for 800 nm field, *α* is azimuthal angle of $\hat{e}$ with respect to the *x*-axis (as shown in Fig. 1), and φ is the relative phase between $E_{\omega }$ and $E_{2\omega }$. In the photocurrent model, THz radiation from the two-color laser filament arises from the transverse electron current *J* driven by the asymmetric *E_L_*. The relation between *J* and *E_L_* is described by

$$\dot{J}(t)+\nu J(t)=\frac{e^{2}}{m_{e}}E_{L}(t)N_{e}(t),$$ (4)

with electron charge *e* and mass *m_e_*. The electron collision frequency *ν* can be neglected in simplified calculations, while the electron density $N_{e}(t)$ in the plasma is calculated using the static tunneling ionization rate described in Ref. 23. The electron current density *J* is two-dimensional since the laser field *E_L_* has two orthogonal components in our experimental condition. We apply the Fourier transform to both sides of Eq. (4) and use the low frequency spectrum of $\dot{J}$ to estimate the intensity of THz emission. Figure 5 shows that the phase retardation of BBO (Γ) strongly affects the ratio of minimum to maximum THz amplitude during 2π radians change of the relative phase φ. In our system, φ is adjusted by translating the position of BBO, and Γ is controlled by tilting the BBO and, thus, varying the effective thickness of the crystal. Hence, the two-dimensional transverse current model has provided a qualitative explanation on the trend observed in Fig. 4.

**FIG. 5.**
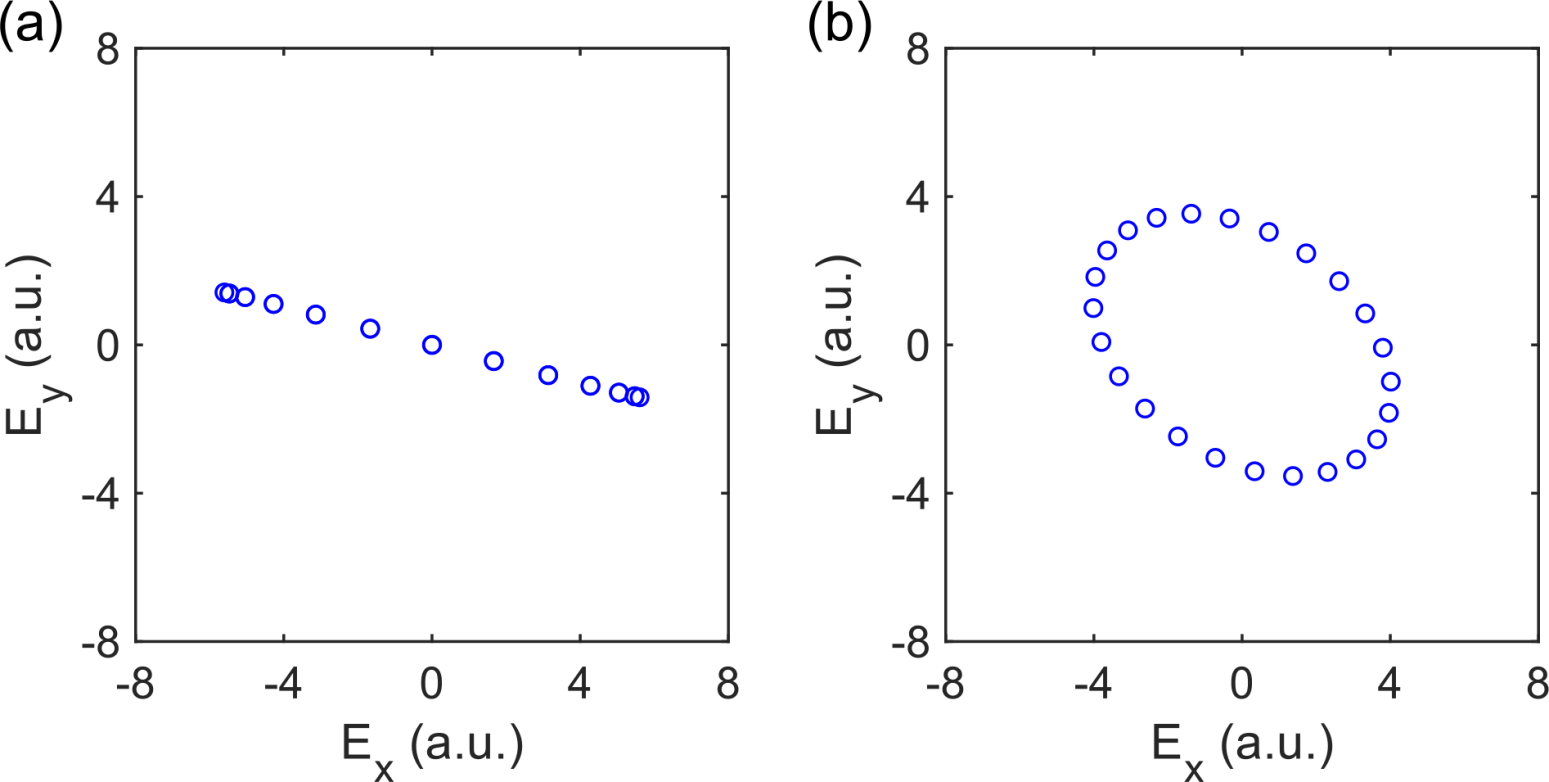
The simulation results confirm the dependence of the THz polarization angle and amplitude on the relative phase φ when (a) Γ=0° and (b) Γ=90°. Γ is the phase retardation of the 800 nm field between $\hat{e}$ and $\hat{o}$ directions.

To demonstrate the broadband capability of the THz-TDP system, we first measured the anisotropic properties of crystal quartz. A 3.0 mm thick A-cut quartz crystal (Newlight Photonics) was tested in the transmission mode to obtain the optical parameters for both the e- and o-axes concurrently in one TDP measurement. The sample was kept at the certain azimuthal angle such that its o-axis would be parallel to the laboratory *y*-direction. Figure 6(a) shows that the phonon resonance at 3.9 THz of the o-axis can be resolved in the transmission spectrum of *E_y_*, while *E_x_* does not show this feature. The extracted refractive indices for the e- and o-axes in the sample are presented in Fig. 6(b), which are in agreement with the previous Fourier-transform infrared spectroscopy (FT-IR) measurement.^24^

**FIG. 6.**
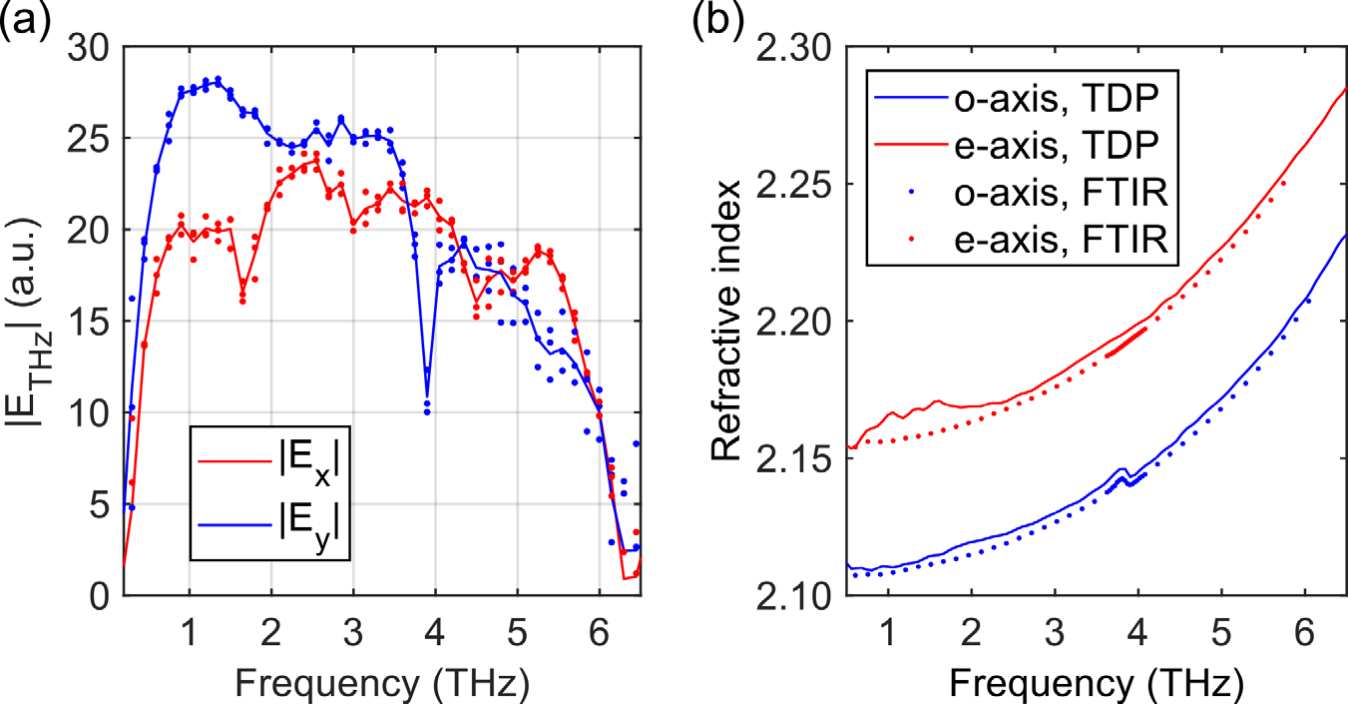
(a) Spectra for the THz waves transmitted through a A-cut quartz crystal. The o-axis of quartz is aligned with the component *E_y_* (blue), while the e-axis is aligned with *E_x_* (red). The extracted refractive indices of the e- and o-axes (b) are similar to the FT-IR measurements described in Ref. 24.

As a second TDP demonstration, we performed reflection measurements on a natural calcite sample. This sample was designed as a group velocity dispersion (GVD) compensation plate (Eksma Optics) with the thickness of 1.7 mm, which exhibits strong absorption above 2 THz.^26^ The crystal's labeled c-axis was oriented such that $E_{x}\;⊥\;c,$ and $E_{y}∥c$. Figure 7 shows that the reflection spectrum of *E_x_* has two peaks, which correspond to the two *E_u_* mode at 3.06 and 6.68 THz. Meanwhile, the spectrum of *E_y_* contains only one peak, which corresponds to the $A_{2u}$ mode at 2.76 THz. We have calculated the theoretical reflection spectra of calcite using the Lorentz oscillation parameters reported in Ref. 25, which shows excellent agreement with the broadband TDP measurements.

**FIG. 7.**
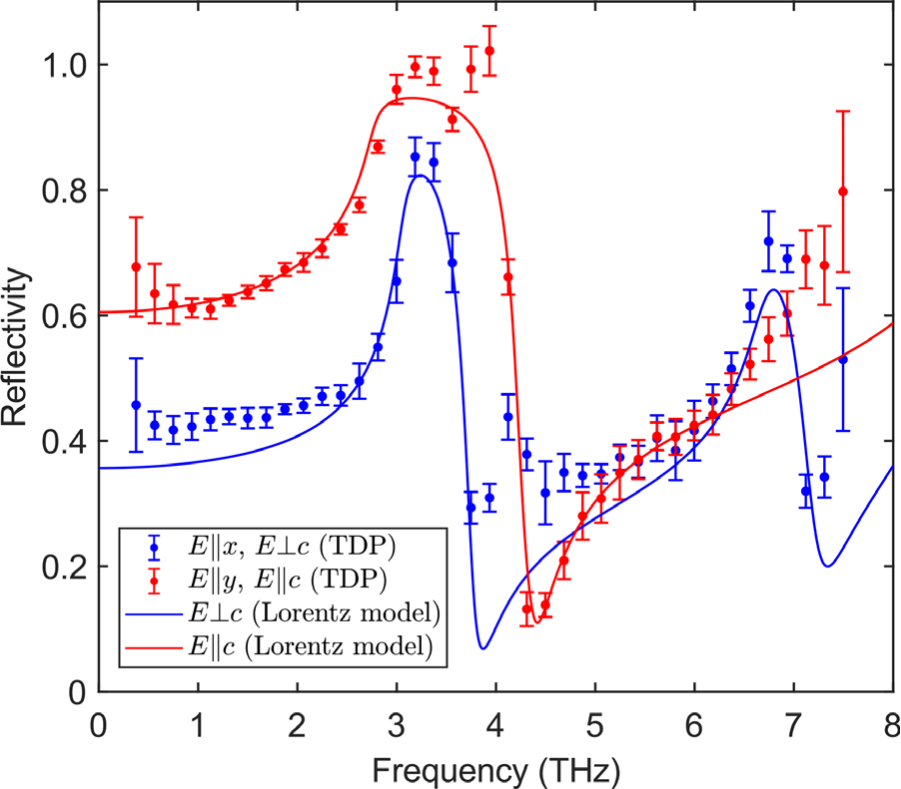
Reflection spectra of calcite ( 45° angle of incidence) for polarization components *E_x_* and *E_y_* compared with the calculations using Lorentz oscillator parameters from Ref. 25.

In conclusion, we have demonstrated a broadband THz-TDP system by using the air plasma as the source and the GaP crystal as the detector. We showed that the polarization angle of THz pulses can be extracted at a fixed time delay with a precision of approximately 1.6° and the frequency-dependent rotation angle with a precision of about 1.9° between 0.7 and 7 THz. We have investigated the polarization control of THz waves generated in two-color laser filaments as a function of the position of BBO and its tilt angle. When rotating the polarization, we observed that the oscillation in the THz amplitudes can be compensated by the BBO tilt angle. This phenomenon was explained by the phase retardation induced by the BBO crystal and a two-dimensional photocurrent model. Subsequently, we have measured the transmission spectra of quartz and the reflection spectra of calcite to demonstrate the extended bandwidth of our system. This THz-TDP system provides a unique approach for broadband polarization measurements in the THz regime.

## Data Availability

The data that support the findings of this study are available from the corresponding author upon reasonable request.

## References

[1] N. Matsumoto , T. Hosokura , T. Nagashima , and M. Hangyo , “ Measurement of the dielectric constant of thin films by terahertz time-domain spectroscopic ellipsometry,” Opt. Lett. 36, 265–267 (2011). 10.1364/OL.36.000265 21263521

[2] K. Wiesauer and C. Jördens , “ Recent advances in birefringence studies at THz frequencies,” J. Infrared, Millimeter, Terahertz Waves 34, 663–681 (2013). 10.1007/s10762-013-9976-4

[3] D. K. George , A. V. Stier , C. T. Ellis , B. D. McCombe , J. Černe , and A. G. Markelz , “ Terahertz magneto-optical polarization modulation spectroscopy,” J. Opt. Soc. Am. B 29, 1406–1412 (2012). 10.1364/JOSAB.29.001406

[4] A. Ferraro , D. C. Zografopoulos , M. Missori , M. Peccianti , R. Caputo , and R. Beccherelli , “ Flexible terahertz wire grid polarizer with high extinction ratio and low loss,” Opt. Lett. 41, 2009–2012 (2016). 10.1364/OL.41.002009 27128061

[5] M. Mičica , V. Bucko , K. Postava , M. Vanwolleghem , J.-F. Lampin , and J. Pištora , “ Analysis of wire-grid polarisers in terahertz spectral range,” J. Nanosci. Nanotechnol. 16, 7810–7813 (2016). 10.1166/jnn.2016.12556

[6] A. Filin , M. Stowe , and R. Kersting , “ Time-domain differentiation of terahertz pulses,” Opt. Lett. 26, 2008–2010 (2001). 10.1364/OL.26.002008 18059761

[7] H. Makabe , Y. Hirota , M. Tani , and M. Hangyo , “ Polarization state measurement of terahertz electromagnetic radiation by three-contact photoconductive antenna,” Opt. Express 15, 11650–11657 (2007). 10.1364/OE.15.011650 19547525

[8] A. Hussain and S. R. Andrews , “ Ultrabroadband polarization analysis of terahertz pulses,” Opt. Express 16, 7251–7257 (2008). 10.1364/OE.16.007251 18545430

[9] K. Peng , D. Jevtics , F. Zhang , S. Sterzl , D. A. Damry , M. U. Rothmann , B. Guilhabert , M. J. Strain , H. H. Tan , L. M. Herz , L. Fu , M. D. Dawson , A. Hurtado , C. Jagadish , and M. B. Johnston , “ Three-dimensional cross-nanowire networks recover full terahertz state,” Science 368, 510–513 (2020). 10.1126/science.abb0924 32355027

[10] P. C. M. Planken , H. K. Nienhuys , H. J. Bakker , and T. Wenckebach , “ Measurement and calculation of the orientation dependence of terahertz pulse detection in ZnTe,” J. Opt. Soc. Am. B 18, 313–317 (2001). 10.1364/JOSAB.18.000313

[11] N. C. J. van der Valk , W. A. M. van der Marel , and P. C. M. Planken , “ Terahertz polarization imaging,” Opt. Lett. 30, 2802–2804 (2005). 10.1364/OL.30.002802 16252780

[12] R. Zhang , Y. Cui , W. Sun , and Y. Zhang , “ Polarization information for terahertz imaging,” Appl. Opt. 47, 6422–6427 (2008). 10.1364/AO.47.006422 19037370

[13] N. Nemoto , T. Higuchi , N. Kanda , K. Konishi , and M. Kuwata-Gonokami , “ Highly precise and accurate terahertz polarization measurements based on electro-optic sampling with polarization modulation of probe pulses,” Opt. Express 22, 17915–17929 (2014). 10.1364/OE.22.017915 25089412

[14] N. Yasumatsu , A. Kasatani , K. Oguchi , and S. Watanabe , “ High-speed terahertz time-domain polarimeter based on an electro-optic modulation technique,” Appl. Phys. Express 7, 092401 (2014). 10.7567/APEX.7.092401

[15] Y. Deng , J. A. McKinney , D. K. George , K. A. Niessen , A. Sharma , and A. G. Markelz , “ Near-field stationary sample terahertz spectroscopic polarimetry for biomolecular structural dynamics determination,” ACS Photonics 8, 658–668 (2021). 10.1021/acsphotonics.0c01876

[16] N. Yasumatsu and S. Watanabe , “ Precise real-time polarization measurement of terahertz electromagnetic waves by a spinning electro-optic sensor,” Rev. Sci. Instrum. 83, 023104 (2012). 10.1063/1.3683570 22380076

[17] K. Xu , E. Bayati , K. Oguchi , S. Watanabe , D. P. Winebrenner , and M. Hassan Arbab , “ Terahertz time-domain polarimetry (THz-TDP) based on the spinning E–O sampling technique: Determination of precision and calibration,” Opt. Express 28, 13482–13496 (2020). 10.1364/OE.389651 32403822 PMC7340380

[18] K. Y. Kim , J. H. Glownia , A. J. Taylor , and G. Rodriguez , “ Terahertz emission from ultrafast ionizing air in symmetry-broken laser fields,” Opt. Express 15, 4577–4584 (2007). 10.1364/OE.15.004577 19532704

[19] J. M. Dai , N. Karpowicz , and X. C. Zhang , “ Coherent polarization control of terahertz waves generated from two-color laser-induced gas plasma,” Phys. Rev. Lett. 103, 023001 (2009). 10.1103/PhysRevLett.103.023001 19659200

[20] Q. Wu and X.-C. Zhang , “ 7 terahertz broadband GaP electro-optic sensor,” Appl. Phys. Lett. 70, 1784–1786 (1997). 10.1063/1.118691

[21] H. Wen and A. M. Lindenberg , “ Coherent terahertz polarization control through manipulation of electron trajectories,” Phys. Rev. Lett. 103, 023902 (2009). 10.1103/PhysRevLett.103.023902 19659205

[22] T. I. Oh , Y. S. You , and K. Y. Kim , “ Two-dimensional plasma current and optimized terahertz generation in two-color photoionization,” Opt. Express 20, 19778–19786 (2012). 10.1364/OE.20.019778 23037030

[23] P. B. Corkum , N. H. Burnett , and F. Brunel , “ Above-threshold ionization in the long-wavelength limit,” Phys. Rev. Lett. 62, 1259–1262 (1989). 10.1103/PhysRevLett.62.1259 10039624

[24] E. E. Russell and E. E. Bell , “ Measurement of the optical constants of crystal quartz in the far infrared with the asymmetric Fourier-transform method,” J. Opt. Soc. Am. 57, 341–348 (1967). 10.1364/JOSA.57.000341

[25] W. J. Tropf , “ Calcium carbonate, calcite (CaCo_3_),” in *Handbook of Optical Constants of Solids*, edited by E. D. Palik ( Academic Press, Burlington, 1997), pp. 701–715.

[26] I. C. Ho and X.-C. Zhang , “ Application of broadband terahertz spectroscopy in semiconductor nonlinear dynamics,” Front. Optoelectron. 7, 220–242 (2014). 10.1007/s12200-014-0398-2

